# Genome Instability in Development and Aging: Insights from Nucleotide Excision Repair in Humans, Mice, and Worms

**DOI:** 10.3390/biom5031855

**Published:** 2015-08-13

**Authors:** Diletta Edifizi, Björn Schumacher

**Affiliations:** 1 Institute for Genome Stability in Aging and Disease, Medical Faculty, University of Cologne, Joseph-Stelzmann-Str. 26, Cologne 50931, Germany; E-Mail: edifizid@uni-koeln.de; 2 Cologne Excellence Cluster for Cellular Stress Responses in Aging-Associated Diseases (CECAD), Center for Molecular Medicine Cologne (CMMC) and Systems Biology of Aging Cologne, University of Cologne, Joseph-Stelzmann-Str. 26, Cologne 50931, Germany

**Keywords:** Ultraviolet Light (UV), DNA damage, aging, Nucleotide-excision repair (NER), Global-genome nucleotide-excision repair (GG-NER), Transcription-coupled nucleotide excision repair (TC-NER), Cockayne syndrome (CS), somatotropic axis, growth hormone/insulin-like growth factor 1 (GH/IGF1) signaling, longevity

## Abstract

DNA damage causally contributes to aging and cancer. Congenital defects in nucleotide excision repair (NER) lead to distinct cancer-prone and premature aging syndromes. The genetics of NER mutations have provided important insights into the distinct consequences of genome instability. Recent work in mice and *C*. *elegans* has shed new light on the mechanisms through which developing and aging animals respond to persistent DNA damage. The various NER mouse mutants have served as important disease models for Xeroderma pigmentosum (XP), Cockayne syndrome (CS), and trichothiodystrophy (TTD), while the traceable genetics of *C*. *elegans* have allowed the mechanistic delineation of the distinct outcomes of genome instability in metazoan development and aging. Intriguingly, highly conserved longevity assurance mechanisms respond to transcription-blocking DNA lesions in mammals as well as in worms and counteract the detrimental consequences of persistent DNA damage. The insulin-like growth factor signaling (IIS) effector transcription factor DAF-16 could indeed overcome DNA damage-driven developmental growth delay and functional deterioration even when DNA damage persists. Longevity assurance mechanisms might thus delay DNA damage-driven aging by raising the threshold when accumulating DNA damage becomes detrimental for physiological tissue functioning.

## 1. Introduction

Aging is characterized by the declining functioning of tissues and organs and the steadily increased risk of succumbing to aging-associated diseases. Understanding the causes of aging and the mechanisms that determine the aging process remain major scientific challenges, particularly in global societies that are faced by the demographic change with increasing burdens of aging-associated diseases.

While the race for successful offspring generation has exerted significant selection for genes promoting the maintenance of somatic functioning early in life, genes that may positively influence somatic maintenance during late post-reproductive life have been largely ignored by natural selection [1]. The maintenance of the genome is of particular importance, as the loss of genetic information cannot be compensated for. Early in evolutionary history, DNA repair processes were already formed to allow the maintenance of species. The importance of genome maintenance for withstanding the aging process has become particularly evident in a variety of genetic disorders that are caused by heritable mutations in DNA repair genes and are manifested in premature aging in a multitude of tissues [2]. Indeed, DNA lesions are constantly formed amid genotoxic attacks by exogenous sources such as UV light, ionizing radiation, or a variety of chemicals as well as endogenous insults such as reactive oxygen species or metabolic byproducts. One outcome of the constant genotoxic challenges is the increased cancer risk with aging when mutations accumulate as a result of erroneous DNA repair [3]. Despite the highly specialized DNA repair systems that maintain genome stability, a fraction of DNA damage might persist and form obstacles for DNA metabolism. When DNA or RNA polymerases are unable to bypass DNA lesions, cells might enter cellular senescence or even undergo programmed cell death, ultimately leading to loss of tissue integrity and reduced regenerative capacities of stem cell compartments.

## 2. DNA Damage Responses in Living Organisms

### 2.1. DNA Repair Systems

DNA damage comes in many different flavors, depending on the nature of the genotoxic attack. The distinct types of DNA lesions are recognized and removed by highly specialized DNA repair systems. Reactive oxygen species (ROS) are produced during a host of metabolic processes and oxidized bases are rapidly removed by base excision repair (BER). During replication DNA polymerases might insert a false nucleotide. In case the exonuclease activity of DNA polymerases is not sufficient to correct the error, mismatch repair (MMR) scans the newly replicated strand to swipe up mistakes overlooked by the replication machinery. Particularly during rapid DNA synthesis, which, for example, is often required when developmental cell proliferation demands high-speed replication, DNA lesions can be bypassed by translesion synthesis (TLS) polymerases that overcome steric obstacles at the cost of an elevated error rate. DNA double strand breaks (DSBs) pose a serious threat for genome integrity because, during replication, broken chromosomes might be unequally distributed, resulting in aneuploidy, which is strongly associated with malignant transformation but can also drive the aging process [4]. DSBs can be quickly joined by error-prone non-homologous endjoining (NHEJ), by microhomology-mediated endjoining (MMEJ), or by lengthy but accurate homologous recombination (HR) when an undamaged template is available in the S and G2 phases of the cell cycle. Sunlight not only forms the primary energy source for living on earth but has posed a genotoxic threat for most organisms living on the surface of the planet. UV irradiation directly interacts with nucleotides to form pyrimidine 6–4 pyrimidone photoproducts (6-4PPs) and cyclobutane pyrimidine dimers (CPDs). Some organisms that are highly exposed to sunlight such as plants and even marsupials possess specialized photolyases that can directly revert either one of those lesions by splitting up the bulky bond. Other organisms employ nucleotide excision repair (NER) to excise a stretch surrounding the lesion [5].

**Figure 1:**
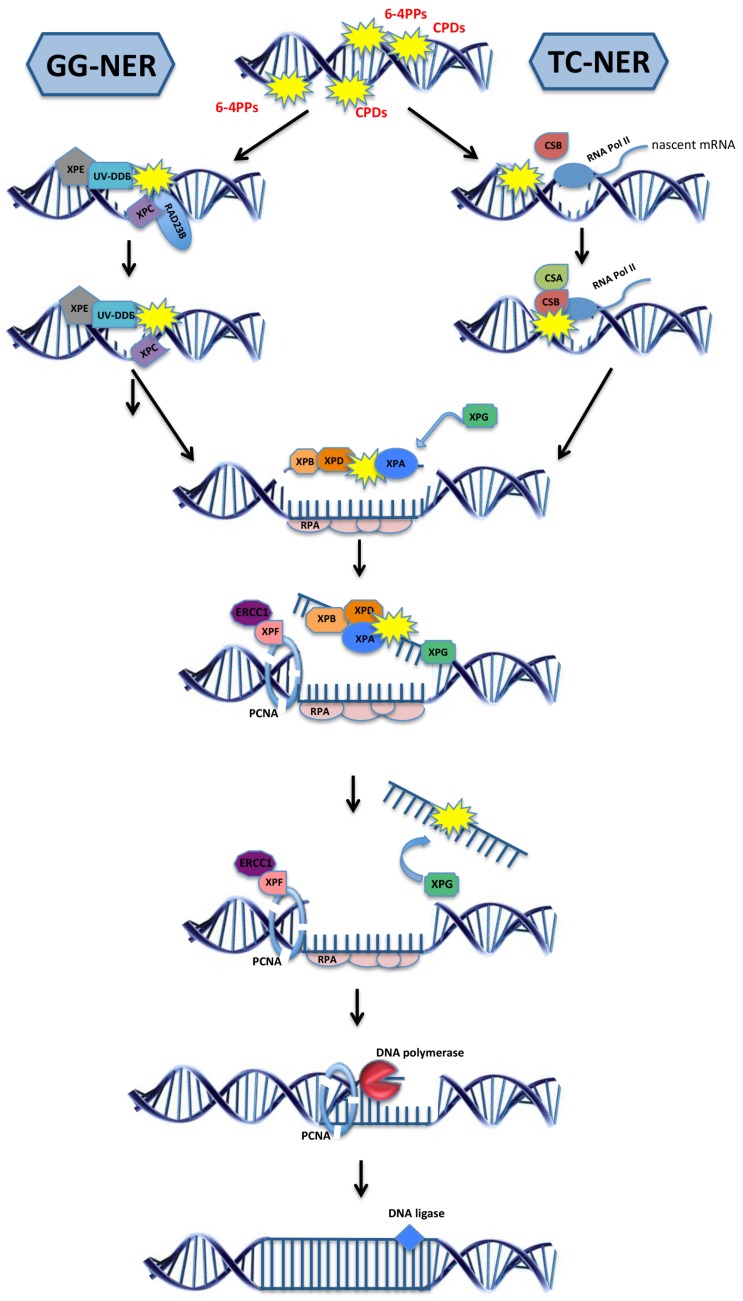
The two sub-pathways of the nucleotide excision repair. Global genome nucleotide excision repair (GG-NER; left) is initiated when the damage sensor proteins XPC and XPE identify helix-distorting lesions throughout the genome. Transcription-coupled NER (TC-NER; right) is initiated by CSB upon the stalling of RNA polymerase II at the site of a lesion, followed by the recruitment of CSA protein. After damage recognition, the TFIIH complex subunits XPB and XPD, together with XPA, are recruited at the lesion site and unwind the helix. The damaged DNA strand is incised by the endonucleases ERCC1, XPF, and XPG while the other single-strand DNA is coated and protected by the protein replication protein A (RPA). DNA polymerases fill the gap and the final nick is sealed by DNA ligases. Over 30 distinct proteins take part in NER and only selected ones are depicted here.

Mutations in any of the above-mentioned DNA repair systems have been identified in human patients who suffer from elevated cancer susceptibility or premature aging syndromes. The cancer types are typically cell-type specific while the functional deterioration is confined to characteristic tissues depending on the specific type of DNA repair defect, and the progeroid (“aging-like”) syndromes are therefore referred to as segmental premature aging. A clear distinction between cancer susceptibility and premature aging has been observed in patient groups that carry distinct mutations in NER. For this reason, mutations in NER have been highly instructive for understanding the links between unrepaired DNA damage and cancer development and accelerated aging, and will be discussed here in further detail. Indeed, clinical phenotypes of highly elevated cancer predisposition or accelerated aging have been linked to mutations that either primarily affect the repair of helix-distorting lesions throughout the genome by global-genome (GG-) NER or specifically in actively transcribed genes by transcription-coupled (TC-) NER (Figure 1) [2].

### 2.2. NER Deficiencies in Humans: Cancer *versus* Development and Aging

The largest group of patients carrying mutations in NER genes suffers from Xeroderma pigmentosum (XP). XP is characterized by pigmentation abnormalities that are intriguingly restricted to the areas of the skin that are exposed to sunlight [6]. The skin can become atrophic and the most deadly XP symptom is the exquisitely high skin cancer susceptibility that was estimated to elevate the risk by several thousand-fold. Rigorous sunlight protection can prevent skin cancer development and majorly improve the livelihoods of XP patients [7]. Particularly, patients that carry a mutation in *XPC* have majorly profited from sunlight protection. However, other XP patients, for instance, those carrying mutations in *XPA*, display additional disease symptoms, namely neurodegeneration. The main difference between those mutations is that the XPC protein functions during the initial damage detection through GG-NER, while XPA is involved in the NER core machinery that is also involved upon damage detection by TC-NER.

Mutations that affect TC-NER but leave GG-NER intact cause Cockayne syndrome (CS), which could hardly be more distinct from XP even though the same types of lesions cannot be repaired just simply in active genes instead of genome-wide. TC-NER is initiated once the RNA polymerase II complex stalls at a lesion and the CSB protein alarms NER through attracting the CSA protein to the lesion whereupon the same NER core complex is assembled [8]. Mutations in *CSB* as well as in *CSA* lead to CS type I which is characterized by severe postnatal growth and mental retardation and accelerated aging, leading to premature death typically at the age of 12-to-16 years [9]. Distinct mutations in those two genes can give rise to even more severe CS type II or even distinct disease syndromes such as cerebro-oculo-facio-syndrome (COFS) or the rather mild UV-sensitivity syndrome (UVSS), generally with as-of-yet unclear genotype-phenotype relations.

Adding even more to the complexity, mutations in NER factors such as *XPD* that are employed by both GG- and TC-NER can lead to XP (typically with neurodegenerative components), rare cases of XP combined with CS, as well as trichothiodystrophy (TTD), where patients suffer from growth and mental retardation and premature aging, in addition to displaying typical brittle hair and nails that have been directly linked to transcription defects resulting from *XPD* or *TTDA* mutations [10]. Indeed, NER proteins not only function during the removal of DNA lesions but also during normal transcription, for example, at the initiation step of RNAPII transcription [11]. How overlapping or functionally distinct the roles during unperturbed transcription or transcription that is compromised by blocking DNA lesions are is currently subject to intense research [12].

### 2.3. Model Organisms as a Tool to Study NER-Deficiency Syndromes

**Figure 2:**
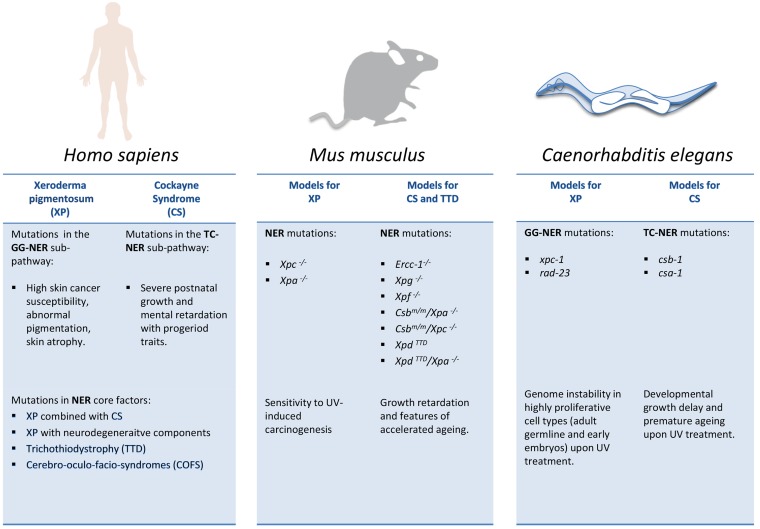
NER mutations in cancer susceptibility, developmental failure, and premature aging. Comparison of distinct consequences of NER mutations in human patients and NER models in mice and *C*. *elegans*. Note that in contrast to human patients, genetic inactivation of GG-NER (e.g., *Xpc*) or TC-NER (*Csb* or *Csa*) genes alone in mice have comparatively mild consequences and mainly elevate susceptibility to UV-induced carcinogenesis. However, when genetically combined, the mutations give rise to severe CS-like phenotypes such as retarded postnatal developmental growth and features of premature aging.

The vast complexity of disease syndromes that are caused by mutations in NER genes has warranted the need for simpler model systems to investigate the consequences of unrepaired DNA lesions during development and aging as well as in the cancer process (Figure 2). Mouse models, often carrying the same point mutations as identified in CS or TTD patients, have enabled drawing important links between disease mutations and underlying physiological alterations to pathological outcomes. However, the mouse disease models for NER syndromes have also proven rather complex, and simpler organisms might clarify cause-effect relations and thus lead to a clearer understanding of the consequences of DNA damage in the developing and the aging organism.

#### 2.3.1. NER Mutant Mice: Linking DNA Damage to Aging and Longevity

In 1990, several mouse models carrying mutations in NER genes were generated. The complete inactivation of NER activity rendered *Xpa* mutant animals highly susceptible to UV-induced carcinogenesis [13]. Mutations not only in *Xpc* but also in *Csb* led to elevated, UV-induced skin cancer susceptibility, indicating distinct consequences compared to human patients in whom skin cancer susceptibility had been primarily linked to GG-NER [14,15]. The recapitulation of the CS- or TTD-associated disease phenotypes had been first observed in mouse models carrying mutations in *Ercc1* [16] or *Xpg* [17], as well as in animals that carried a TTD-associated mutation in *Xpd* whose progeroid features were greatly enhanced by an additional mutation in *Xpa* [18]. Additionally, the mild phenotypes of *Csb* mutant mice were severely enhanced when either *Xpc* or *Xpa* were additionally inactivated [19]. In contrast, *Ercc1* or *Xpg* mutations alone already resulted in severe growth retardation and premature death within three weeks after birth. The rather complex outcome of the mouse genetics indeed indicated that the repair defect alone might not sufficiently explain the disease etiology as, for instance, the lack of *Xpa* alone abolishes all measurable NER activity, while the single mutant mice only display elevated, UV-induced skin cancer incidence [13,19]. Alternative explanations have suggested that the response to oxidative damage might be compromised by specific NER mutations [20] or that defects in the transcription of developmental genes might be accountable for the severe growth defects observed in the mutant combinations [21,22]. In addition, recent metabolic profiling has suggested that depletion of mitochondrial NAD^+^ resulting from PARP activation caused by defective NER might lead to energy depletion, ultimately contributing to degenerative phenotypes [23].

Studies that comprehensively assessed gene expression profiles of mice that carried distinct mutations in NER genes provided important insights into physiological alterations in mice suffering from growth retardation and accelerated aging. Consistent with their developmental growth defects, genes that regulate the somatic growth or the somatotropic axis were already dampened in young *Ercc1^−/−^*, *Csb^m/m^/Xpa^−/−^* double, and *Xpd^TTD^/Xpa^−/−^* double mutants [19,24,25]. The dampening of the somatotropic axis has also been observed in progeroid mouse models that are associated with elevated genome instability, albeit independent of NER, including *Sirt6^−/−^* mice as well as the *Zmptse24* knockout animals that model Hutchinson-Gilford Progeria Syndrome (HGPS) [26,27]. The consequences of an attenuated somatotropic axis can be non-cell-autonomous, as evidenced in Sirt6*^−/−^* mutants as well as in telomerase-deficient animals. For instance, the defects of hematopoietic stem cell (HSC) engraftment were dependent on the progeroid mutation in the host animals but not on the respective mutation in the transplanted HSCs [26,28], suggesting that the alterations in the endocrine environment could hamper the regenerative potential of stem cells.

Intriguingly, the somatotropic axis is intricately linked to the aging process itself. The organismal growth is promoted by insulin-like growth factor-1 receptor (IGF-1R) pro-mitotic signaling. The ligand IGF-1 is secreted to a large extent by the liver but also by other peripheral tissues when they are stimulated by growth hormone receptor (GHR) signaling, while GH itself is secreted by the pituitary gland [29]. Ames and Snell dwarf mice are unable to develop a pituitary gland and display severely retarded body growth similar to *Ghr* knockout mice, suggesting that among the pituitary hormones, GH is the critical somatic growth stimulus [30]. Strikingly, Ames and Snell dwarf mice as well as *Ghr* knockout animals are not only small in size but also extraordinarily long-lived. While *Igf1r* knockout is lethal, heterozygote females were reported to have an extended lifespan [31]. While constitutive dampening of the somatotropic axis evidently leads to lifespan extension in mice, naturally aging animals also show reduced GH and IGF-1 levels in their circulation [29]. Indeed, somatotropic attenuation appears to be a common feature of prematurely aging animals early in their lives, normative aging animals at old age, and mice with extended longevity throughout their lifespan [32]. It is conceivable that the dampening of the somatic growth axis might exert protective functions [2]. However, in the case of the DNA repair-deficient animals, the shift toward tissue maintenance at the expense of growth might be employed too early in their lives and, instead of protecting the animals, impedes their developmental growth. Indeed, IGF-1 treatment could overcome the early death of *Zmpste24* knockout animals [27]. The protective effect of a dampened somatotropic axis has been observed in several lines of evidence. For example *Igf1r^+/−^* heterozygous animals showed elevated protection from oxidative stress that was induced by paraquat injections [31]. Specific knockout of the *Igf1r* in the brain was cytoprotective in an Alzheimer disease model [33,34]. The mitogenic function of IGF-1 signaling has been established to promote cancer cell growth and, indeed, Ames dwarf as well as *Ghr* knockout mice showed protection from fatal neoplastic lesions [35,36]. It is likely that the consequences of somatotropic attenuation suppress the tumor development in CS, TTD, and XFE mouse models. Indeed, even *Xpd^TTD^* mutant animals that display relatively mild premature aging phenotypes compared to *Ercc1^−/−^* or *Csb^m/m^/Xpa^−/−^* mutant mice developed fewer tumors than age-matched wild-type animals [37].

Mechanistically, it was demonstrated that mammalian cells respond to transcription-blocking DNA lesions by down-regulating GHR and IGF-1R, leading to IGF-1 resistance and elevated cellular resistance to oxidative stress [38]. When the DNA lesions persist, such as in *Ercc1^−/−^* single or *Csb^m/m^/Xpa^−/−^* double mutant cells, somatotropic attenuation becomes permanently installed. Although the mouse models are highly instructive when assessing pathologies resulting from DNA repair defects, the physiological alterations, particularly those occurring during developmental growth, are exquisitely complex. Therefore, simpler metazoan systems might be utilized to shed new light on the response mechanisms to unrepaired DNA lesions in the context of development and aging.

#### 2.3.2. *C*. *elegans* as a Metazoan Model for NER Deficiencies

The nematode *Caenorhabditis elegans* has been an important model system for investigating a range of biological processes and has gained considerable fame when its developmental lineage was revealed [39], apoptosis as a genetically controlled cell death was identified [40], and gene knockdown by RNA interference was established [41]. *C*. *elegans* undergoes a deterministic developmental program that leads to the hatching of L1 larva that subsequently pass through the L2, L3, and L4 larval stages to form an adult animal that, in case of hermaphrodites, self-fertilizes to generate offspring within the first few days of adulthood. In adult worms all somatic tissues are postmitotic and cannot be regenerated. Only the germline contains mitotically dividing germ cells that, as they are driven out by the Notch-mediated germ stem cell niche, differentiate through meiosis while they are gradually pushed towards the spermatheca where the oocytes are fertilized. Missegregation of chromosomes during meiosis, for instance, at elevated temperatures, leads to occasional loss of an X chromosome, consequently resulting in the generation of male progeny.

The first radiation-sensitive variants of *C*. *elegans* were already isolated during the 1980s [42]. The specific investigation of DNA damage responses were facilitated by the discovery of physiological cell death in adult animals that, in contrast to the previously known developmental cell death, occurred in a rather dynamic fashion, specifically in meiotic pachytene cells [43]. In late stages of meiotic pachytene, the meiotic recombination intermediates ought to have been completely processed, suggesting that the apoptotic response might be involved in the demise of germ cells bearing unresolved recombination intermediates. Indeed, ionizing radiation (IR) treatment steeply elevated the number of apoptotic corpses, verifying that DSBs could trigger cell death in the pachytene cells [44]. IR-induced apoptosis was then quickly determined to involve similar DNA damage checkpoint mechanisms that were known already from yeast studies and are conserved all the way to humans. The *C*. *elegans* p53-like *cep-1* was determined to trigger apoptosis in response to DNA damage by transcriptionally inducing the BH3-only domain proteins EGL-1 and CED-13 which then activate the apoptosome [45,46,47,48]. CEP-1 specifically triggers apoptosis in the late meiotic pachytene stage as, only upon reaching this stage of meiosis, the GLD-1-mediated repression of *cep-1* mRNA is alleviated and prevents uncontrolled apoptotic demise as long as normal meiotic recombination is under way [49].

In contrast to the germ cells, the postmitotic somatic tissues are highly radioresistant and withstand IR doses of up to 1000 Gray without significant reduction of lifespan [50]. The radioresistance of somatic tissues might be due to the fact that IR-induced DSBs will only rarely hit and affect an actively transcribed gene. Indeed, in contrast to DSB-inducing IR, both somatic and germ cells display considerable sensitivity to UV irradiation [51]. The NER genes are highly conserved between worms and humans and a complete inactivation of NER renders the nematodes exquisitely sensitive to even low doses of UV [52,53]. Mutations in the two NER branches result in intriguingly distinct outcomes of the UV response. GG-NER mutations lead to UV sensitivity, specifically in highly proliferative cells in the germline and early embryos [53], while TC-NER inactivation renders postmitotic tissues hypersensitive to the same genotoxic insult [54]. Indeed, the exposure of young TC-NER-deficient larvae to even low doses of UV results in severe growth retardation. Even when, upon UV exposure, TC-NER-defective *csb*-*1* or *csa*-*1* mutant larvae are permanently arrested in their somatic development, the germ cells continue their mitotic expansion [54,55]. In contrast, UV-treated GG-NER-deficient *xpc*-*1* or *rad*-*23* mutant larvae complete somatic development after passing through a transient delay similar to wild-type animals, though they are unable to develop a germline. Completely NER-deficient *xpa*-*1* or *csb*-*1*;*xpc*-*1* double-mutants arrest both somatic and germline growth at even lower UV doses [54]. When adult GG-NER-defective animals are exposed to UV irradiation, mitotically proliferating cells in the germ stem cell compartment exhibit chromosome bridges and form DSBs, likely resulting from replication fork breakdown amid persistent CPD and 6-4PP lesions. Taken together, the phenotypic distinction of GG- and TC-NER mutants has established *C*. *elegans* as a useful genetic model where GG-NER defects lead to genome instability in proliferating cells that in humans comprises a causal event in malignant transformation, while TC-NER defects lead to severe developmental growth delays mirroring a primary CS-associated clinical feature. In addition, adult TC-NER mutants prematurely succumb with failing tissue functioning when they experience UV treatment.

The tissue-specific DNA damage sensitivity allows for investigating responses throughout the entire animal to the presence of genome instability in specific cell types. For instance, somatic tissues develop highly elevated resistance to oxidative and heat stress in response to the persistence of UV-induced DNA lesions in the germline of GG-NER deficient mutants [56]. The same “germline DNA damage-induced systemic stress resistance” (GDISR) was then observed upon various germline-specific DNA damage paradigms and even endogenous meiotic DSBs were sufficient enough to trigger stress resistance throughout the somatic tissue of the animals. Mechanistically, GDISR is instigated by the activation of an innate immune response through MAPK signaling in the damaged germ cells that then leads to the elevated activity of the ubiquitin proteasome system (UPS) in somatic tissues that in turn enhances the stress resistance [56]. The enhanced somatic endurance might allow the animals to extend their reproductive lifespan to facilitate offspring generation once the DNA damage in the genomes of the germ cells has been repaired and the DNA damage checkpoint-mediated germ cell arrest has been alleviated [57].

The conserved NER mechanisms acting during somatic development and tissue maintenance provide a unique opportunity to investigate DNA damage response mechanisms during metazoan development and aging. Similar to the somatotropic attenuation that was observed in the progeroid NER mutant mice, insulin/insulin-like signaling (IIS) also responds to transcription-blocking lesions in *C*. *elegans*. In the nematode, *daf-2* encodes the sole homolog for the IGF-1R and the related insulin receptor (IR). Mutations in *daf-2* and the downstream PI3 kinase *age*-*1* were the first genetic mutations to be identified that conferred extended longevity [58,59]. The lifespan extension of those mutants is completely reverted when, in addition, the FOXO transcription factor *daf-16* is mutated [58]. The observation that mutations in single *C*. *elegans* genes could extend lifespan established that longevity was regulated by genetic mechanisms and thus formed an important basis for modern aging research. The critical effector of lifespan extension in IIS mutants is the constitutive activation of DAF-16 [60]. Under normal conditions, DAF-16 is hyperphosphorylated by IIS signaling and sequestered in the cytosol. Only when IIS is turned off the hypophosphorylated DAF-16 enters the nucleus and regulates gene expression. A plethora of DAF-16 target genes have been identified, some of which regulated ROS scavengers, detoxification enzymes, chaperones, and a large number of functionally uncharacterized genes [61,62]. IIS is active in nutritious environments that are usually found under laboratory conditions but are probably less frequent in natural habitats. Under starvation conditions, IIS is switched off, leading to activation of DAF-16. During development, DAF-16 activation leads to developmental arrest, and under certain conditions even to the formation of alternative *dauer* larvae that can survive for extended periods of time. Once a nutritious environment is encountered, the arrested larvae resume development, advance to adulthood and produce offspring. Interestingly, in recent sampling experiments of natural *C*. *elegans* populations, growing and reproducing animals were exclusively isolated on fresh compost heaps and decaying fruits where UV irradiation might actually pose a natural hazard [63].

Similar to starvation, DAF-16 responds in somatic tissues upon UV treatment. However, in contrast to the consequences of DAF-16 activity in the starvation response, in the presence of UV-induced DNA lesions, DAF-16 promotes developmental growth even when CPD and 6-4PP lesions persist due to defective NER [54]. Specifically upon UV-induced DNA damage, DAF-16 induces developmental growth genes that under starvation conditions are repressed by the FOXO transcription factor. The specificity was determined to depend on the recognition of GATA promoter sites by the GATA transcription factor EGL-27, which together with DAF-16 promotes resilience to UV-induced DNA lesions. In adult animals, DAF-16 activity prevents the functional decline of tissues and extends the lifespan even in completely NER-deficient *xpa*-*1* animals that were exposed to UV irradiation. However, with advancing age, the responsiveness of DAF-16 specifically to DNA damage fades, thus ultimately rendering the animals defenseless against the functional decline resulting from the lesions [54].

Taken together, the *C*. *elegans* studies on NER mutants have established system-wide response mechanisms to genome instability that leads to physiological adaptations, namely the extension of the reproductive lifespan when the genome integrity of germ cells is compromised. In somatic tissues IIS attenuation, which is similar to that in mammals, triggered upon transcription-blocking DNA lesions culminates in the activation of DAF-16, which overcomes the developmental delay and promotes tissue integrity in the presence of unrepaired DNA lesions. The *bona fide* longevity assurance mechanisms of DAF-16 activity might antagonize DNA damage-driven growth retardation and aging by elevating tolerance towards persistent or accumulating DNA damage, thus raising the threshold when the age-dependent accumulation of DNA damage leads to functional deterioration.

## 3. Conclusions

Human syndromes that are caused by mutations in NER genes are rare genetic disorders that can have debilitating consequences. Mutations in distinct NER genes and even distinct mutations in the same NER gene can lead to diverse clinical phenotypes ranging from elevated skin cancer susceptibility in XP patients to growth retardation and premature aging in children suffering from CS. While the lives of some XP patients could be greatly improved by rigorous protection from sunlight, there is no cure available for any of the NER-associated syndromes. Mouse models have provided important insights into the physiological consequences of NER mutations. Intriguing links to endocrine adjustment have been established, which suggest that genetic pathways of longevity regulation respond to unrepaired DNA damage. Recent studies in *C*. *elegans* have complemented the work in mammalian disease models and revealed how adaptations to genome instability could overcome growth retardation and maintain the functional integrity of aging tissues. It will be highly interesting to explore how adaptations through the IIS/somatotropic axis could alleviate pathology and might antagonize DNA damage-driven aging in humans as well.
